# 

**Published:** 1816-06

**Authors:** 


### CORRESPONDENCE.

Mr. Johnson on the Topography of New Orleans, in answer to a paper in the Edinburgh Journal for April on the same subject, in our next.

The Title and Index to this Volume will be given in our succeeding Number.

---

END OF VOL. I.

### INDEX.

ABSCESS of the liver burſting into the lungs 182

Acid, pruſſic, caſe of poiſoning by 84

Acids, concentrated, powerful poiſons 511

——— ——— treatment of poiſoning by 513

Adheſion of wounded parts, how accompliſhed 524

Adheſive ſtraps, obſervations on the application of 340

African ſlaves, on certain diſeaſes of 373

Amauroſis of both eyes, caſe of 468

——— ——— completely cured 469

Anaſtomosing aneuriſm in the hand, caſe of 541

Anatomy of the head and neck 50, 234, 315

Aneuriſm of the heart, caſe of 295

——— of the ſubclavian artery, operation for 319

——— of the carotid artery 320

——— from anaſtomoſis, deſcription of 327

——— operation for by the French ſurgeons 332

——— of the cœliac trunk, caſe of 359

——— of the left orbit cured by tying the carotid 478

——— Axillary, cured by tying the artery below the clavicle 480

Animal œconomy, action of arſenious acid upon 428

Antimonial poiſons, effects of, and antidotes againſt 502

——— medicines unſafe for children 161

Anus, artificial, ſpontaneouſly cured 540

Apoplexy, conſequent on aneuriſm of both auricles of the heart 278

Apothecaries' Hall, courſe of examination at 184

Arſenical poiſons, varieties of 425

——— ſolution decompoſed by the galvanic pile 452

——— paſte, fatal effects from the application of 335

Arſenious acid, chemical hiſtory of 425

——— ——— ſymptoms of poiſoning by 429

Arteriotomy, mode of performing 330

Aſcarides in the rectum, how to be removed 410

Aſphyxia, diſſertation on 80, 168

——— from ſuffocation 168

——— from drowning 169

——— from ſtrangulation 170

Aſphyxia, from nitrogen gas 171

——— from want of reſpirable air 171

Babington, Mr. Aſſiſtant Surgeon to the Lock Hoſpital 368

Bailey's, Mr. H. caſe of contracted inteſtine 106

Biography, medical 535

Biſmuth, phyſiological action of 511

——— treatment of poiſoning by 511

Bladder and its appendages, on the diſeaſes of the 486

Bleeding, the neceſſity of, in local inflammation 379

Blood, on the cauſes of the motion of the 515

Blood-letting, experiments on 88, 441

——— unſucceſsful in hydrophobia 533

Blue diſeaſe, caſe and diſſection of 451

BOOKS, NEW, ANNOUNCED. — Dr. Halliday on the Doctrine of Incitement, 95. M. P. J. Roux's Viſit to London in 1814, 185. The Second Part of Orfila's Syſtem, ib. Dr. Armſtrong's Practical Illuſtrations of Febrile Diſeaſes, ib. Dr. Male on Forenſic Medicine, ib. Dr. Stewart's Obſervations on Uterine Hæmorrhage, ib. Mr. Whateley's Practical Obſervations on Ulcers of the Legs, 2d Edit. 290. Captain Laſkey's Account of the Hunterian Muſeum, ib. Dr. Adams's Memoirs of John Hunter, 368. Mr. Armiger's Rudiments of the Anatomy and Phyſiology of the Human Body, 544. Mr. Howſhip's Practical Obſervations on Diſeaſes of the Urinary Organs, ib.

BOOKS REVIEWED. — Sir James Fellowes's Reports of the Peſtilential Diſorders of Andaluſia, 21. Dr. Parry's Elements of Pathology and Therapeutics, 37, 148, 205. Mr. Allan Burns on the Anatomy of the Head and Neck, 50, 234, 315. Mr. Highmore's Caſe of a Fœtus found in the Abdomen of a Young Man, 68. Mr. Hill, on the Nature and Cure of Inſanity, 118, 250. Mr. Croſs's Sketches of the Medical Schools of Paris, 129, 254, 331. Dr. Clarke's Commentaries on the Diſeaſes of Children, 160, 221. Dr. Willan's Treatiſe on Porrigo and Impetigo, 296. Dr. Yeats on the early Symptoms of Water in the Brain, 307. Dr. Farre on the Morbid Anatomy of the Liver, 311, 392. Mr. Hey's Treatiſe on Puerperal Fever, 381. Mr. Manſfield

Clarke on Difeafes of Females, attended by difcharges, 398, 492. M. Orfila's Treatife on Poifons derived from the three Kingdoms of Nature, 413, 501. Sixth volume of Medico-Chirurgical Tranfactions, 471. Mr. Wadd's Cafes of difeafed Bladder and Tefticle, 486. Dr. Carfon on the Caufes of the Motion of the Blood, 515.
Bronchocele, defcription and treatment of 245
Bruce's, Mr. cafe of wounded œfophagus 369
Bulam fever, obfervations and remarks on 199, 449
——— remarks on 97
——— contrafted with the Batavian endemic 98
——— a difeafe, *sui generis*, attacking the human frame but once 103
Bullet found in the fubftance of the brain 92
Burns, Mr. on the anatomy of the head and neck 50, 234, 315
Burder, Dr. Phyfician to the Weftminfter General Difpenfary 368
Cadiz, medical topography of 22
——— fever, origin and progrefs of 24
Calculus, biliary, large, fpontaneous difcharge of 477
Calvert, Dr. on the plague at Malta 471
Cancer, defcription of French practice in 334
Cancerous tumor in the ftomach, cafe of 289
Carcinoma recti, fymptoms of 411
——— treatment of 412
——— uteri, defcription of 493
——— treatment of 494
Cardiac affection, cafe of 179
———, hepatic, and pulmonic difeafe, cafe of 290
Carotid artery tied for the cure of aneurifm in the orbit 478
Carfon, Dr. on the caufes of the motion of the blood 515
Cafe of poifoning by pruffic acid 84
Cafes of the utility of evacuations and low regimen 2
Caftration, mode of performing, at Paris 333
———, fingular confequences from the operation of 366
Catheters, gum-elaftic, French method of making 288
Chamberlayn's, M. cafe of axillary aneurifm 480
Chemical hiftory of corrofive fublimate 417
Chemiftry, a knowledge of the minutiæ of, not effential to a medical practitioner 87
Chevalier's, Mr. cafe of croup, cured by tracheotomy 484
Circulation of the blood, how effected 521
Clarke, Mr. Mansfield, on difeafes of females, attended by difcharges 398
Cœliac trunk, cafe of aneurifm of the 359
Converfion of difeafes, remarks upon 212
Convulfions, chronic, in children, treatment of 232
——— in children, defcription of 164
——— treatment of 166
Coppery poifons, varieties of, and effects on the animal fyftem 504
Correfpondence, 95, 188, 290, 368, 456, 544
Croup, cured by the operation of tracheotomy 484
Curry, Dr. on fudden death 196
———, on the effects of the eau medicinale 198
Cutaneous affections, treatment of 337
——— affection, fingular cafe of 338
Cynanche laryngea, fuccefsfully treated 482
Dalrymple's, Mr. cafe of aneurifm 478
Davis, Dr. J. B. Phyfician to the Univerfal Difpenfary for Children 544
Death, fudden, occafioned by biliary obftruction 197
——— produced by a blow on the ftomach 276
——— fudden, cafe of, with unufual fymptoms 542
——— apparent, from fumes of fea-coal 179
Dentition, proper treatment during 162
Defcription of the fœtus found in the abdomen of a young man 74
Diagnofis between central ftricture of the ftomach and other abdominal lefions 281, 288
Difeafes of children, commentaries on 160, 221
Diffection of a fatal case of tubera diffufa 395
——— of a patient poifoned by opium 437
——— juridical, mode of conducting 528
Diffections, minutes of, 181, 292, 361, 367, 289
*Docimasie pulmonaire*, article from the French Dictionary of Medical Sciences 348
Doughty, Mr. on the yellow or Bulam fever 449
Dropfy, a common termination of inflammation 46
——— of the maxillary finus, cafe of 534
——— final caufe of 151

### INDEX.

Dupuytren's, M. cafe of a fœtus found in a boy 69
Dyfentery, fymptomatic of worms and fcurvy 375
——— cafe of 115
——— fuccefs of mercurial treatment in 116
Dyfpepfia, pathology of 156
Earle's, Mr. cafe of retention of urine 476
Eau medicinale, deleterious effects of 198
Electricity, Letters on the fubject of, 91, 177, 362
Ellis, Mr. on the dyfentery of African flaves 375
Emetic tartar, fymptoms of poifoning by 503
Epilepfy, the caufes of explained 208
Eryfipelas endemic at Rio Janeiro 463
Evacuations, on the utility of, in fome obfcure difeafes 1
Examination, juridical, of a corpfe, mode of conducting 526
Excifion of two of the metacarpal and carpal bones 541
Eye, cafe of malignant difeafe of the 329
Farre, Dr. on the morbid anatomy of the liver 341
Fellowes, Sir James, on the peftilential diforders of Andalufia 21
Females, on the difeafes of, attended by difcharges 398
Fever, Weft Indian, cafes of 18
———, at Cadiz, origin and progrefs of 24
——— defcription of 26
Fiftula lachrymalis, operation for 328
Fœtus found in the abdomen of a young man 68
——— found in the abdomen of a child 479
Fracture of the fkull, cafe of 276
Fractures, great attention paid to, in the French hofpitals 258
French Dictionary of Medical Sciences, 80, 168, 262, 348, 525
Galvanic pile, decompofition of arfenical folution by the 452
Gilham, Mr. Surgeon to the Univerfal Difpenfary for Children 544
Gold, muriate of, action on the human fyftem 510
——— treatment of poifoning by 510
Golding, Mr. letter from, to the Editors 91
Gray, Mr. on difeafes of African flaves 373
Hæmorrhage, pathology of 148
——— final caufe of 151
Hæmorrhoid's accompanying mucous difcharges in females 409
Hare lip, defcription of, and operation for 328
Heart, cafe of fatal lefion of the 272
Hepatic affection, cafes of 113, 290
Hernia, French mode of operating in 256
——— crural, containing the uterus, ovaries, and part of the vagina 538
——— congenita, on the caufes of 491
Hey, Mr. on puerperal fever 381
Highmore's, Mr. cafe of a fœtus found in a young man 68
Howfhip's, Mr. cafe of difeafed omentum 109
Hunterian Mufeum, an account of publifhed 290
Hydrencephalus, early fymptoms of 308
——— fymptoms of the advanced ftage of 311
——— treatment of 312
——— cafe of, with diffection 367
Hydrocele endemic at Rio Janeiro 467
Hydrophobia, cafe of 531
——— unfuccefsfully treated by blood-letting 533
Hydroftatic trial of the lungs, objections to 348
Hydrothorax, cafe of, with obfcure fymptoms 452
Hyflop's, Mr. cafe of incontinence of urine 477
Idiotifm in children, caufes of 231
Ileum, cafe of fphacelated 271
Impetigo, divifion of, into fpecies 303
——— treatment of the various fpecies of 306
Infantile difeafes, obfervations on 339
Inflammation, on the various terminations of 41
——— final caufe of 151
Inflation of the lungs of new-born children, cafes of 352
Infanity, cafes of 251
——— on the nature and cure of 118
——— fthenic and afthenic forms of 120
——— always dependent upon corporeal difeafe 119
——— predifpofing and exciting caufes of 126
Inteftine, contracted, cafe of 106
Inverfio uteri, defcription and treatment of 408
Jaundice, extenfive prevalence of 188
Johnfon, Mr. in reply to Dr. Yeats 470
——— cafe of malformation 202
——— cafe of cardiac difeafe 290
——— on the utility of evacuations and low regimen 1
Juridical inquiry into the caufes of death, mode of conducting 526
Kennedy's, Dr. cafe of poifoning by nitric oxyd of quickfilver 189

Larrey's, M. cafe of paralyfis of the mouth and throat 450
Lafkey's, Captain, account of the Hunterian Mufeum 290
*L'Ecole de médecine*, defcription of 133
LECTURES ANNOUNCED.—At St. Bartholomew's Hofpital, 95. Dr. Clutterbuck on Medicine, 95, 456. Mr. Taunton on Anatomy and Surgery, 95, 544. At St. Thomas's and Guy's Hofpitals, 184. Dr. Merriman on Midwifery, 185. Mr. Fox on the ftructure and Difeafes of the Teeth, 290. Mr. Carpue on Anatomy and Surgery, 544
Ligature of the carotid artery, cafe of 177
Liver, abfcefs of the, burfting into the lungs 182
—— inquiry into the morbid anatomy of the 341
Locked jaw, cured by oil of turpentine 473
London yearly bill of mortality 96
—— Vaccine Inftitution, report of, 453
Lungs, trial of, to afcertain whether a child has breathed 262
—— Plocquet's method 265
—— Daniel's method 265
M'Lleod, Mr. on the Bulam fever 198
Malformation, curious cafe of 202
—— of the urinary and genital organs 180
Mamma, cancerous, cafe of operation for 334
Maxillary finus, cafe of dropfy of the 534
Medical Annuitant and Benevolent Society 94, 183
—— additional table by the 289
—— permanent eftablifhment of the 543
—— topography of Cadiz 22
—— mifcellanies, 87, 176, 272, 362, 441, 535
Medical Schools of Paris, fketches of 129, 254, 331
Medicina Nautica 17, 111
Medico-Chirurgical Tranfactions, review of 471
Medicus, letter from 87
Meeting of army and navy furgeons 456
Melancholia, hiftory of 124
Memoir on a cafe of poifoning by opium 435
Menuret de Chambaud Jean Jacques, biographical fketch of 537
Metaftafis, curious inftance of 277
Meteorological Regifter for Harwich 186
Mode of detecting poifon by corrofive fublimate 421
Momentum of the blood, caufe of various difeafes 215
Mortality, London yearly bill of 96
Nicotiana, on the ufe of, in retention of urine 476
Nitric oxyd of quickfilver, deleterious effects of 190
—— acid, peculiar effects of 513
Nervous conftitution, defcription of 205
Newcaftle, memorial of practitioners at, on the apothecaries' bill 367
Obituary, 95, 185, 290, 368, 456, 544
O'Brien's, Mr. cafe of removal of the human fpleen 8
Obfervations on the late act of parliament for examination at Apothecaries' Hall 183, 367
—— on difeafes at Harwich 188
Odier's, Dr. letter to Dr Curry on a cafe of fudden death 197
Œfophagotomy, mode of performing 249
Œfophagus, wounded, cafe of, with diffection 369
Omentum, difeafed, cafe of 109
Operation, extraordinary 91
—— for the removal of an enlarged fcrotum 475
Opiates extremely prejudicial to children 161
Opium, cafe of poifoning by 435
Orfila, M. on animal, vegetable, and mineral poifons 413
Origin and progrefs of the Cadiz fever 24
Oxymuriate of mercury, chemical hiftory of 417
Palmer, Dr. on the central ftricture of the ftomach 281
—— cafe of contracted ftomach 10
—— cafe of extirpation of the parotid gland 457
Pancreas, difeafed, diftinguifhed from central ftricture of the ftomach 287
Paralyfis, incomplete, of mouth and throat from a wound penetrating the cranium 450
—— in children, caufes of 231
Pariera, Dr. on the medical topography of Rio Janeiro 463
Paris, Sketches of the Medical Schools of 129, 254, 331
Parotid gland, cafe of excifion of 457
Parry's, Dr. elements of pathology and therapeutics 37, 148, 205
Pathology of the cervical fafcia 234
—— and therapeutics, elements of 37, 148, 205
—— of the tongue, tonfil, and falivary glands 323
Pessaries, different kinds of, and mode of applying 405
Pettigrew, Mr. Surgeon to the Univerfal Difpenfary for Children 544

Phillips's, Dr. cafe of locked jaw cured by oil of turpentine 473
——— cafe of a fœtus found in the abdomen of a child 479
Phrenitis in children, obfervations on 221
——— caufes of 225
——— treatment of 226
Plague, on the origin of the, at Malta 471
Plan for the extirpation of the venereal difeafe 93
—— of examination at Apothecaries' Hall 184
Poifons, treatife on 413
——— corrofive, action and fymptoms of 415
——— mercurial, treatment in cafes of 193
Polypus of the uterus, fymptoms of 494
Porrigo, divifion of, into fpecies 296
Powers's, Mr. cafes of Weft Indian fever 17
Preffure employed to cure incontinence of urine 477
Private practitioners of medicine in France, claffed 130
Procidentia uteri, fymptoms of 402
——— treatment of 403
——— veficæ, fymptoms and treatment of 406
——— vaginæ, caufes and cure of 407
Profluvium vaginale, varieties of 400
Pruffic acid, a virulent poifon 84
Puerperal fever, treatife on 381
——— hiftory and fymptoms of 382
——— method of cure in 385
——— cafes of 387
Purgatives, choice of, in hydrencephalus 309
——— cafe of extirpation of the parotid gland 457
Purging, utility of, in puerperal fever ib.
Pym's, Mr. obfervations on the Bulam fever 97
Reaction, exemplification of the falutary effects of 247
Regimen, very low, ufeful in certain obfcure difeafes 1
Reports of the peftilential diforders of Andalufia 21
Reviewer's anfwer to Dr. Yeats 470
Rheineck's, Mr. cafe of amaurofis 468
Rheumatifm, efficacy of electricity in 362
Rio Janeiro, on the medical topography of 463
Roberts's, Dr. cafe of cynanche laryngea 482
Royal Society, officers of the, elected 92
Salivary glands, pathology of the 324
Scrotum, enlarged, fuccefsfully removed 474
Sea-coal, apparent death from the fumes of 179
Secretions, natural, from the uterus 399
Seeds, Dr. on Blood-letting 88, 441
Selections, 80, 168, 262, 348, 435, 525
Silver, nitrate of, phyfiological action of 509
——— treatment of poifoning by 510
Sketches of the Medical Schools of Paris 129, 254, 331
Skull, fractured with depreffion, cafe of 276
Small-pox nearly exterminated in Paris 339
Spanifh practice in fevers, fpecimens of 31
Spleen, human, removal of the, without injury 8
Stomach, obfervations on the central ftricture of the 281
——— contraction of, near its centre 10
——— cafe of ulcerated fcirrhus of 285
Students of medicine in Paris, claffification of 133
Sudden death, cafe of, with unufual fymptoms 254
Surgeons, army and naval, meeting of the 456
Sympathies of the fexual organs 401
Symptoms of poifoning by arfenious acid 429
Syphilis, efficacy of mercurial falts in 336
Tenon, Jacques, biographical fketch of 536
Tefticle, on the difeafes of the 489
Teftis, difeafed, cafe of 333
Thomas's, Mr. cafe of biliary calculus 476
Thymus gland fubject to fcrofulous enlargement 242
Thyroid gland, pathology of the 243
——— gland enlarged in cardiac difeafes 181
Tin, fymptoms of poifoning by 508
Titly's, Dr. cafe of enlarged fcrotum 474
Tongue, pathology of the 323
Topography, medical, of Cadiz 22
——— of Rio Janeiro 467
Toxicology, a general fyftem of 413
Tracheotomy, defcription of the operation of 315
——— employed for the cure of croup 484
Tranfactions, Medico-Chirurgical, review of 471
Treatife on Poifons derived from the three kingdoms of nature 413
Treatment of a patient poifoned by corrofive fublimate 423

Treatment of a patient poisoned by arsenious acid 433

Tubercle, fleshy, of the uterus, description and symptoms of 495

Tubera diffusa, varieties and symptoms of 392

——— circumscripta, description of 343

————— symptoms accompanying 343

——— diffusa, character and symptoms of 344

Tumors about the throat, description of 235

Turpentine, oil of, useful in locked jaw 473

Ulcerated scirrhus of the stomach, case of 285

Umbilical tumor, case of 365

Urine, retention of, relieved by nicotiana 476

——— incontinence of, cured by pressure 477

Urethral stricture, treated by the conical silver catheter 260

Uterine discharges from increased action 496

————— debility 498

Uterus, natural secretions from the 399

——— diseases of, attended by discharges 402

——— cancer of the, description of 492

————— treatment of 494

——— polypus of the, symptoms and cure of 494

——— fleshy tubercle of, symptoms of the 496

Uwins, Dr. Physician to the City Dispensary 92

Vaccination, improved method of securing 277

————, practice of the improved mode of 365

Venereal disease, plan for the extirpation of the 93

Verdigris, chemical history of 504

——— symptoms arising from the poison of 505

——— the best antidotes against 506

Vertigo, explanation of the causes of 208

*Vis a tergo* not the sole cause of venous circulation 518

Von Embden, Dr. communication from 468

Wadd's, Mr. cases of diseased bladder and testicle 486

Wadsell, Mr. Extra Surgeon to the Universal Dispensary for Children 544

Walker, Dr. John, extract of a letter from 289

West India fever, cases of 18, 111

Willan, Dr. on porrigo and impetigo 296

Yeats, Dr. in answer to the review of his book 377

——— on bleeding in local inflammation 379

——— on the early symptoms of water in the brain 307

Yellow bark, obtained from the cinchona hirsuta 92

Zinc, preparations of, given in epilepsy 233

——— action of, on the animal œconomy 508

——— treatment of poisoning by 508

PRINTED BY W. THORNE, RED LION COURT, FLEET STREET.

